# The role of pharmaceutical industry in building resilient health system

**DOI:** 10.3389/fpubh.2022.964899

**Published:** 2022-12-01

**Authors:** Kanika Saxena, Sunita Balani, Pallavi Srivastava

**Affiliations:** ^1^Amity Business School, Amity University, Lucknow, Uttar Pradesh, India; ^2^Human Research Management and Organizational Behaviour, Jaipuria Institute of Management, Lucknow, Uttar Pradesh, India

**Keywords:** pharmaceutical, pandemic, sustainable development goals, one health, joint external evaluation (JEE), health security

## Abstract

**Objectives:**

This study explores the interrelationship among the current sustainability agenda of the pharmaceutical industry, based on the United Nation sustainable development goals (SDGs), the elements of the Joint External Evaluation (JEE) tool, and the triad components of the One Health approach.

**Methods:**

A cross-walk exercise was conducted to identify commonalities among SDGs, JEE assessment tool, and One Health approach. An in-depth study of 10 global pharmaceutical firms' corporate sustainability reports and COVID-19 response plan for 2019–2020 was also conducted.

**Results:**

The result of the exercise showed the existence of a direct and indirect relationship among the SDGs, elements of JEE assessment tool, and One Health approach. For example, both no poverty (SDG 1) and zero hunger (SDG 2) are linked with food safety targets under the JEE and with human and animal health under the One Health approach.

**Conclusion:**

This study adds a new dimension emphasizing the possibility of tailoring the pharmaceutical industry's activities under the sustainability agenda to strengthen global health security while remaining consistent with the One Health approach.

## Introduction

In the era of globalization and advancement of information technology, diseases such as Ebola virus disease (EVD), Zika virus, and COVID-19 continue to pose a threat to the world. The EVD epidemic in West Africa (2014–2016) is a lesson to be learned when it comes to identifying the key measures needed to boost public health and prevent future outbreaks. A disease outbreak causes death, loss of livelihood, community upheaval, and wider socioeconomic ramifications. The recent COVID-19 pandemic has caused not only severe disruption of normal daily life but also an increase in unemployment and mental illness (1). It has emphasized not only the importance to build up a resilient health system but also the animal–human interface facilitating transmission of zoonotic microorganisms (2). The World Health Organization recommends each country or member state to develop a National Action Plan for Health Security (NAPHS) tailored to their needs after identifying priority areas through the Joint External Evaluation (JEE) assessment tool when it comes to health security under the International Health Regulation (3, 4). The NAPHS should be based on a multi-sectoral approach to improve identified priority areas and should involve all stakeholders. When it comes to developing the NAPHS, the pharmaceutical industry could be a key stakeholder. The ongoing COVID-19 pandemic and vaccine development underpin the pharmaceutical industry's critical role in global health security. Furthermore, it emphasizes the need for both animal and human health to avoid disease transmission. This resonates with the One Health approach which covers human, animal, and environment under one umbrella (5). Even though the pharmaceutical sector has a direct impact on human, animal, and environment, its role in achieving universal health coverage and building resilient health system remains undefined and the responsibility lies mainly on government agencies (6). In this study, we aimed to explore whether the activities adopted by the pharmaceutical companies as part of their sustainability agenda can directly or indirectly contribute to achieving set targets in the JEE assessment tool, thereby contributing aid in the development of the NAPHS to increase global health security and address the three components of the One Health approach (7).

## Literature review

When it comes to corporate social responsibility (CSR) and sustainability, the pharmaceutical business has always been scrutinized. Smith emphasized the incorporation of CSR in the business strategy to have a competitive advantage (8). Although the pharmaceutical business recognizes CSR and sustainability, its initiatives are criticized as profit-driven rather than health-oriented. The government has resisted involving pharmaceutical industries in the development of health policies due to public opposition. However, post-COVID-19, the public opinion about the pharmaceutical industry has changed opening new opportunities to collaborate and work together in building resilient health system (9). Additionally, eminent research groups like Marques et al. advocated the inclusion of health as the fourth pillar of sustainability (10). Similarly, Buse and Hawkes emphasized the inclusion of health in the sustainable development goals in 2015 (11). However, there is a need to identify the activities under the sustainability agenda which promote good health, secure animal–human–environment interface avoiding transmission of zoonotic disease, and increase health security.

## Current trends in moving toward building resilient health system

Building resilient health system has become a priority following COVID-19. The recent Global Health Security Conference 2022 emphasized resilient health systems (12). Under the leadership of the director general, the WHO began building health emergency preparedness, response, and resilience (HEPR) architecture in March 2022 (13). To better prepare for upcoming emergencies, a call for private–public collaboration has been made. “Pharmaceuticalisation of security” a term quoted by Elbe in 2014 was pioneer research showing the critical role of pharmaceuticals in providing security for well-being of the society (14). The pharmaceutical industry's contribution to fostering health security is recognized globally, and national legislation has been created particularly to regulate the sector (15). Also, a study to analyze the pharmaceutical industry's role in the Nigerian health security found that government legislation needs to enforce ethical standards and socially responsible manufacturing (16). In another study by Mugwagwa and Banda, the significance of public–private collaboration in creating health systems showed the importance of local pharmaceutical suppliers/manufacturers in low–middle-income nations (17). The new European strategy also emphasizes the pharmaceutical sector's vitality to boost pandemic preparedness. The new approach aims to develop strong supply chains and assure ongoing raw material and drug deliveries during public health crises (18).

### Global health security agenda (GHSA) and national action plan for health security (NAPHS)

Post-EVD most countries joined the GHSA an initiative started by the USA in collaboration with other international organizations to improve global health security as required under the umbrella of International Health Regulation (4, 19). The WHO recognized the necessity of NAPHS in its NAPHS for All handbook, and all nations were asked to participate voluntarily and build a similar strategy for a resilient health system (20). To develop a NAPHS for a country, the GHSA advised an initial external country assessment process of the current health security framework to identify the areas of improvement in the health sector. This external country assessment process was further developed by the WHO in collaboration with other partners to develop the Joint External Evaluation (JEE) tool (21). The JEE assessment tool helps to identify the areas for improvement in reference to global health security which further aids in the development of the NAPHS for health security (22, 23).

Additionally, the sustainable development goals (SDGs) advised by the United Nations Global Compact aim to provide a comprehensive approach to building a healthy and secure environment, therefore providing basic support to increase global health security (24). Though there are a number of studies available on the SDGs assessment, NAPHS, JEE, etc., none of them shows the cross-walk among the SDGs, JEE assessment tool, and One Health.

### Sustainability in pharmaceutical sector and its linkage with pandemics

Sustainability has been one of the main agendas during the last decade globally and is known to add business value by tracking company's ability to manage resources in an intelligent manner. Pharmaceutical companies drive the sustainability agenda under the corporate social responsibility and report their sustainability initiatives against the 17 SDGs (**Table 2**) (25, 26). The pharmaceutical sector research agenda is an interesting example where the leadership drive directed their research toward COVID-19 and successfully developed potential vaccines which underwent clinical trials, thereby proving that the pharmaceutical sector is an important stakeholder in building resilient health system.

It is also important to note that the pharmaceutical industry encounters challenges when it comes to aligning their activities with the 17 SDGs. These challenges include a lack of effective leadership, budgetary restrictions, harmonious partnerships, exhaustiveness and complexity of interlinkages among the goals and their targets, and a lack of monitoring and evaluating methods for assessing the progress of implementation (27).

### Sustainable development goals (SDGs), health security, and one health approach

The COVID-19 pandemic led to increased awareness about health security and the One Health approach. The study by Dixon showed the necessity of global system changes in the setting of the SDGs as per emerging needs to the COVID-19 pandemic (28). Similarly, Ottersen and Engebretsen interpreted COVID-19 as a challenge to current SDGs and argued that the pandemic has shown the challenges confronted when it comes to universal health coverage (29). In addition to it, the COVID-19 pandemic highlights the necessity to include the One Health approach in both sustainability and the development of health security to avoid future pandemics (30). According to the CDC, “One Health is a collaborative, multi-sectoral, and transdisciplinary approach—working at the local, regional, national, and global levels—with the goal of achieving optimal health outcomes recognizing the interconnection between people, animals, plants, and their shared environment” (31). Considering the threat and origin of the recent pandemic, it is utmost necessary to address emerging zoonotic diseases and antimicrobial resistance under the One Health approach to build up health security (32). The triad components of the One Health include human health, animal health, and the environment. Although the One Health approach has been known for a century, its cross-linking with sustainability and health security remains unexplored.

### Gap analysis

Based on the literature review, a gap analysis was conducted to identify the critical findings followed by the development of the research questions (33). Table 1 shows the important gaps identified after an in-depth study of the literature. Additionally, backward and forward referencing technique was used to trace the limited literature on this subject (34). Forward referencing is the technique of finding the newest articles, books, or book chapters that cite a particular article, book, or book chapter by looking at “cited by” on Google Scholar. This search looks forward in time to determine how this article is contributing to the scholarly conversation. We may stay up to date with the most recent research on a certain subject by using forward referencing. On the contrary, backward referencing is the technique of looking for all the references that are cited in a single article. This search looks backward in time to see what led to the article you start with. This is an important activity as it enables one to determine the reasons why gaps exist in the article they are currently reading. “Ascendancy searches” and “descendancy searches” are other terms for forward and backward citation tracing, which is basically searching up and down for citations, respectively (34).

**Table 1:** Important publications and identified research gaps, sustainable development goals, one health, India, 2019–2021.

**S. No**.	**Author**	**Key findings**	**Research gap**
1.	Bali and Taaffe (19)	(i) Both sustainable development goals and global health security agenda are exclusive but do complement each other (ii) Synergy can build between the two if the implementers work together	Areas showing synergies not presented at the granular level
2	Hotez (35)	(i) Synergies could be built up between sustainable development goal 3 (good health and wellbeing) and global health security agenda to identify activities which can improve disease surveillance and strength health system	Ground-level activities still not marked or linked with sustainable development goals
3.	Kajee et al. (36)	Many sustainable development goals show relationship with developmental origin of health and diseases	Indirect linkage could have been investigated
4	Kickbusch et al. (37)	There is a need to global health security risk to be addressed under sustainable development goals	Direct and indirect linkage between global health security and current sustainable development goals analysis not present
5	Sinclair (38)	Highlights the importance of human–animal–environment interface in building health security	Direct and indirect linkage among sustainable development goals, health security, and One Health approach not presented

### Rationale behind the study

The preceding literature review justifies the need for an in-depth study into the relationship among sustainability, health security, and the One Health approach to identify commonalities and a point of intersection. To aid this study, the research questions were formed through problematization as recommended by Alvesson and Sandberg (39) and by identifying the following gap—*the relationship among the SDGs, key elements determining health security, and One Health approach remains unclear*.

Based on the gap analysis, the following research questions were formed:

*RQ1. Is there any relationship between the SDGs and development of National Action Plan for Health Security (NAPHS) based on the JEE assessment tool and One Health approach?*

*RQ2. Do the activities under the COVID-19 response plan directly or indirectly contribute to the fight against the pandemic thereby increasing health security?*

RQ^*^–Research Question.

## Methods

To explore the possible solution to the identified gaps, a cross-walk exercise was performed to map the links among the SDGs, key elements of the JEE assessment tool (40), and triad components of the One Health approach (32). The cross-walk exercise showed direct and indirect relationships among the SDGs, elements of JEE, and triad components of the One Health approach. A direct relationship means that a particular SDG fulfills the set targets in the JEE assessment tool (e.g., SDG3—good health and well-being is directly related to immunization) and addressed the One Health approach, whereas an indirect relationship means that an SDG can contribute to achieving a particular set target in the JEE assessment tool to some degree (e.g., SDG4—quality education is necessary for people to understand the importance of immunization) and the One Health approach. The cross-walk exercise (40) helps to show the direct or indirect relationship among the SDGs, JEE, and One Health.

### Process flow

1. The cross-walk exercise was performed by conducting online group discussions with experts from the fields of medicine, pharmaceutical sciences, and management. Two experts were chosen from each area, depending on their knowledge and willingness to participate voluntarily. The experts were asked to link each element in the SDGs, JEE, and One Health with specific reasoning. At the end of the discussion, a consensus was built within the expert group to finalize the cross-mapping. In this study, we used the group discussion technique and post-collating the views of experts build up a consensus within the group to finalize the cross-mapping. Initially, we recorded all the opinions of experts individually for the cross-mapping. Each identified linkage was given a score in terms of how many experts agree to the linkage. Later, the captured linkages were discussed in the group and particularly those ones that scored very less (e.g., “1” meaning only one expert identifying the linkage). A consensus was built upon the final cross-mapping result. This technique is a modified version of nominal group technique (41).
2. *Selection criteria for sample from pharmaceutical industries:* The sample size was confined to 10 considering the requirement of the sample set to conclude significant results (42). The selection of the pharmaceutical companies followed two inclusion criteria. (a) *Geographical location:* To justify the global pharmaceutical sector, the companies were selected to represent Asia, Africa, Europe, and the USA pharmaceutical sector. During the selection from these continents, their ranking in the pharmaceutical executive top 50 list for the year 2020 was considered (43) and, (b) *Contribution to the current pandemic:* Certain companies were included in the study especially if they have made extraordinary contribution, such as development of COVID-19 vaccine.
3. A detailed analysis of corporate sustainability reports of the selected pharmaceutical companies for the year 2019 was conducted to classify the activities under the corresponding SDGs, JEE assessment tool elements, and One Health approach.

## Results

### Cross-walk among SDGs, JEE assessment tool, and one health to identify the capacity building areas in the development of NAPHS

Sustainable development goals (SDGs) as advised by the UN Global Compact (26) are given in Table 2 along with their definitions. The key elements of the JEE assessment tool include prevent, detect, response, IHR-related hazard, and point of entry (POE). The JEE assessment tool is used to assesses country's capacity to prevent, detect, and response to public health threats, such as pandemic and other global health emergencies (21). Table 3 shows subcategories under the key elements of the JEE assessment tool, and Supplementary Table S1 shows their corresponding set targets. The One Health approach triad comprises three components, namely, human health, animal health, and environment (44).

**Table 2:** Sustainable development goals and their definitions^b^^c^, sustainable development goals, one health, India, 2019–2021.

**Number**	**SDGs^a^**	**Definition**
SDG^**a**^ 1	No poverty	End poverty in all its forms everywhere
SDG^**a**^ 2	Zero hunger	End hunger, achieve food security and improved nutrition, and promote sustainable agriculture
SDG^**a**^ 3	Good health and well-being	Ensure healthy lives and promote well-being for all at all ages
SDG^**a**^ 4	Quality education	Ensure inclusive and equitable quality education and promote lifelong learning opportunities for all
SDG^**a**^ 5	Gender equality	Achieve gender equality and empower all women and girls
SDG^**a**^ 6	Clean water and sanitation	Ensure availability and sustainable management of water and sanitation for all
SDG^**a**^ 7	Affordable and clean energy	Ensure access to affordable, reliable, sustainable and modern energy for all
SDG^**a**^ 8	Decent work and economic growth	Promote sustained, inclusive and sustainable economic growth, full and productive employment, and decent work for all
SDG^**a**^ 9	Industry, innovation, and infrastructure	Build resilient infrastructure, promote inclusive and sustainable industrialization, and foster innovation
SDG^**a**^ 10	Reduced inequalities	Reduce inequality within and among countries
SDG^**a**^ 11	Sustainable cities and communities	Make cities and human settlements inclusive, safe, resilient, and sustainable
SDG^**a**^ 12	Responsible consumption and production	Ensure sustainable consumption and production patterns
SDG^**a**^ 13	Climate action	Take urgent action to combat climate change and its impacts
SDG^**a**^ 14	Life below water	Conserve and sustainably use the oceans, seas, and marine resource for sustainable development
SDG^**a**^ 15	Life on land	Protect, restore, and promote sustainable use of terrestrial ecosystems, sustainably manage forests, combat desertification, and halt and reserve land degradation and halt biodiversity loss
SDG^**a**^ 16	Peace, justice, and strong institutions	Promote peaceful and inclusive societies for sustainable development; provide access to justice for all and build effective, accountable, and inclusive institutions at all levels
SDG^**a**^ 17	Partnership for the goals	Strengthen the means of implementation and revitalize the global partnership for sustainable development

a SDG denotes sustainable development goal.

b Please refer to reference number (25) in the reference list: United Nations. Transforming our world: the 2030 agenda for sustainable development (2015). Available at: http://www.un.org/ga/search/view_doc.asp?symbol=A/RES/70/1&Lang=E (accessed January 19, 2021).

c Please refer to reference number (26) in the reference list: Fleming A, Russell MW, Hansen H, Sams L. The sustainable development goals: A case study. Mar Policy (2017) 86:94-103.

**Table 3:** Categories of assessment under key elements in joint external evaluation tool^a^, sustainable development goals, one health, India, 2019–2021.

**Prevent (P)**	**Detect (D)**	**Respond (R)**	**Other IHR-related hazards (O)**
(P1) National legislation, policy and financing (P2) IHR coordination, communication and advocacy	(D1) National laboratory System (D2) Real-Time Surveillance (D3) Reporting	(R1) Preparedness (R2) Emergency Response Operations (R3) Linking public health and security authorities	(O1) Points of entry (O2) Chemical events
(P3) Antimicrobial resistance (P4) Zoonotic disease (P5) Food safety (P6) Biosafety and biosecurity (P7) Immunization	(D4) Workforce development	(R4) Medical countermeasures and personnel deployment (R5) Risk communication	

a Please refer to reference number (21) in the reference list: World Health Organization?. Joint external evaluation tool: International Health Regulations 2005 (2016). Available at: https://apps.who.int/iris/handle/10665/204368 (accessed January 25, 2021).

**Figure 1:**
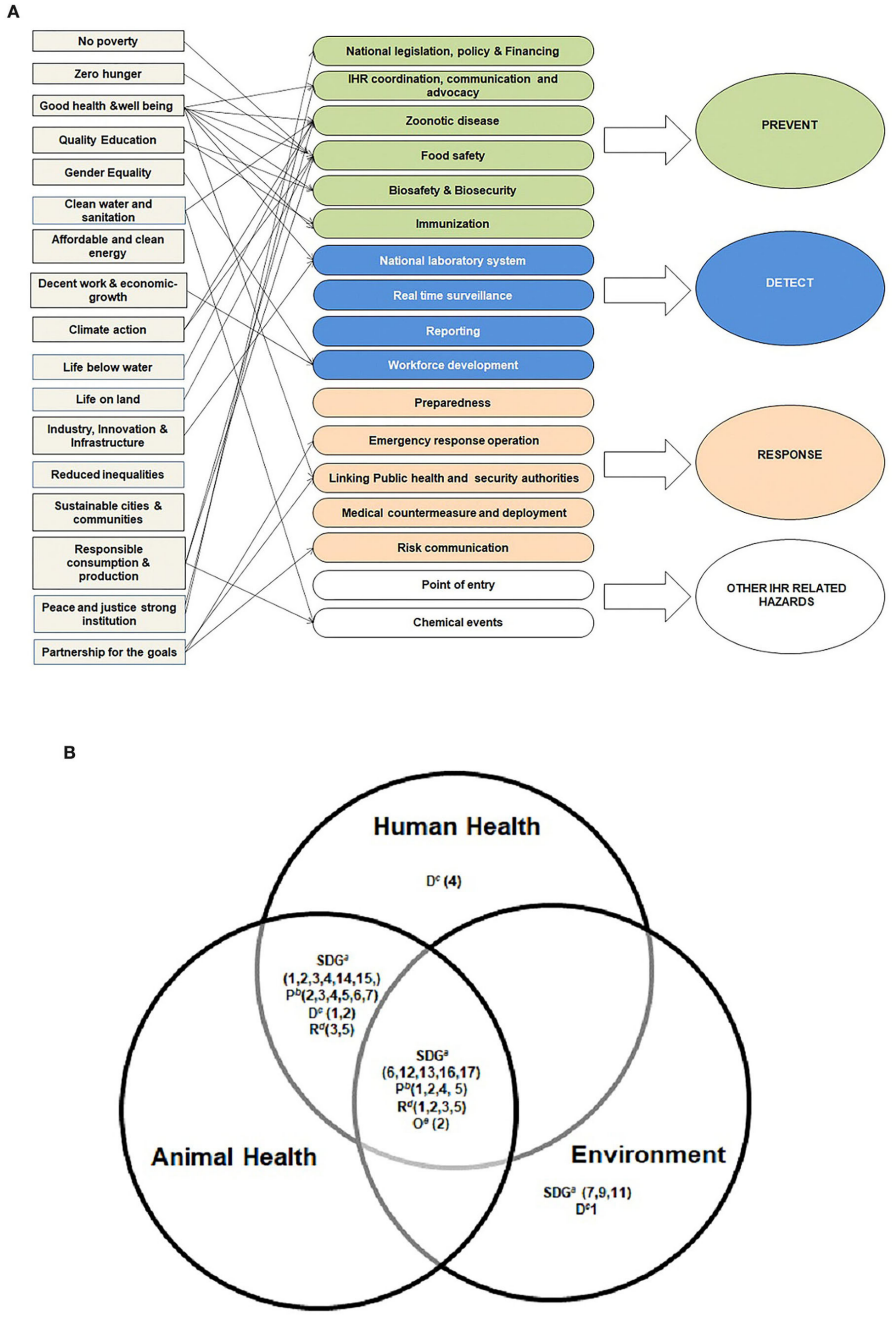
**(A)** Cross-walk between 17 sustainable development goals and joint external evaluation categories, sustainable development goals, one health, India, 2019–2021. **(B)** Cross-walk showing linkage between sustainable development goals, joint external evaluation, and triad components of one health approach, sustainable development goals, one health, India, 2019–2021. ^a^SDG denotes Sustainable Development Goals. ^b^P denotes “prevent” category in joint external evaluation tool. ^c^D denotes “detect” category in joint external evaluation tool. ^d^R denotes “response” category in joint external evaluation tool. ^e^O denotes “other IHR related hazards” category in joint external evaluation tool.

Cross-walk exercise was conducted among the 17 SDGs, various categories under the JEE assessment tool, and triad components of the One Health. Figure 1A shows the direct and indirect relationships between the SDGs and elements of the JEE assessment tool. Figure 1B represents a Venn diagram showing the link among the SDGs, elements of the JEE assessment tool, and triad components of the One Health approach which includes human health, animal health, and environment. The SDGs and the elements of JEE common to all triad components of One Health are represented in the center overlapping region of the three circles. This shows direct and indirect relationships as depicted in Figures 1A,B, respectively.

### Activity mapping for each pharmaceutical company

The recent COVID-19 pandemic has shown the importance of the pharmaceutical industry in confronting a pandemic. Without their significant contribution in particular rapid development and deployment of vaccines, it would have not been possible to overcome it. Additionally, their support to achieve the SDGs cannot be ignored. Among the few to mention are initiatives to improving access to medicine, promoting eco-friendly agricultural practices, distribution of food in the community, and supporting infection, prevention, and control activities, such as hand sanitation in hospital and healthcare settings. Johnson & Johnson developed a PRISMA health device distribution network that allows a single ventilator to support respiratory action in two patients at the same time (45). Pfizer came forward to support the Center of Disease Control (CDC), USA, by creating a specialized SWAT team to provide drug development expertise (46). Eli Lilly adopted an innovative approach such as electronic prescribing to ensure insulin supply to the patient during lockdown phases (47). Bayer has made remarkable steps in supporting farmers and eco-friendly agricultural practices (48). Bharat Biotech in India has been praised for the development of low-cost COVAXIN which has been distributed globally particularly in African nations to overcome the pandemic (54).

The sustainability report for the year 2019 of the 10 sample companies representing Asia, Africa, Europe, and the USA was studied, and the number of activities was mapped on the SDGs along with the corresponding JEE categories under prevent, detect, response, and other IHR-related hazards as given in Table 4.

**Table 4:** Activities under 17 sustainable development goals, joint external evaluation, and one health for the year 2019–2020, sustainable development goals, one health, India, 2019–2021.

**SDGs^a^**	**JEE^b^ categories under prevent (P), detect (D), response (R), and other IHR-related hazards (O)**	**One health approach (Triad—Human health, Animal health, and Environment)**	**Johnson & Johnson**	**Pfizer**	**Eli Lilly**	**Bayer**	**GSK**	**Sanofi**	**AstraZeneca**	**Aspen**	**Sun Pharma**	**Bharat Biotech**
(SDG^**a**^ 1) No poverty	(P5) Food Safety	Human health Animal health										
(SDG^**a**^ 2) Zero hunger	(P5) Food Safety	Human health Animal health				9						
(SDG^**a**^ 3) Good health and well-being	(P2) IHR Coordination, communication and Advocacy (P3) Zoonotic disease (P5) Food safety (P6) Biosafety and biosecurity (P7) Immunization (D1) National laboratory System (D2) Real time surveillance, (R3) Linking public health and security authorities	Human health Animal health	9	4	6	4	14	6	10	9	6	1
(SDG^**a**^ 4) Quality Education	(P6) Biosafety and biosecurity (P7) Immunization	Human health Animal health	6	2	7	5	2	1	2	4	5	1
(SDG^**a**^ 5) Gender Equality	(D4) Workforce development	Human health	2	2	3	3	3		1	2		
(SDG^**a**^ 6) Clean water and sanitation	(P4) Zoonotic disease (O2) Chemical events	Human health Animal health Environment	1		1	1		1	7	5	7	1
(SDG^**a**^ 7) Affordable and clean energy		Environment	4		1					1	4	
(SDG^**a**^ 8) Decent work and economic growth	(D4) Workforce development	Human health	4		4	3		1	5	2	6	3
(SDG^**a**^ 9) Industry, Innovation and infrastructure	(D1) National laboratory system	Environment	1				2				1	
(SDG^**a**^ 10) Reduced inequalities												
(SDG^**a**^ 11) Sustainable cities and communities		Environment	3						3			
(SDG^**a**^ 12) Responsible consumption and production	(P4) Zoonotic disease (P5) Food safety (O2) Chemical events	Human health Animal health Environment	2	6		5	1	2	2	5	8	3
(SDG^**a**^ 13) Climate action	(P4) Zoonotic disease (P5) Food safety	Human health Animal health Environment	1	1				1	2	2	1	
(SDG^**a**^ 14) Life below water	(P4) Zoonotic disease	Human health Animal health				1						
(SDG^**a**^ 15) Life on land	(P5) Food safety	Human health Animal health		1	4			1	1			
(SDG^**a**^ 16) Peace and justice strong institution	(P1) National Legislation, policy and financing (P2) IHR Coordination, communication and advocacy	Human health Animal health Environment										
(SDG^**a**^ 17) Partnership for the goals	(R1) Preparedness (R2) Emergency response operation (R3) Linking Public Health and security authorities (R5) Risk communication	Human health Animal health Environment	13	13	5	5	8	6	9	8	2	
Total no. of activities			46	29	31	36	30	19	42	38	40	9

a Sustainable development goals.

b Joint External Evaluation.

Interesting results were found after the classification of the activities under the corresponding SDGs, JEE, and One Health approach. Johnson & Johnson, AstraZeneca, and Sun Pharma lead in taking the initiatives to fulfill the sustainability goals. However, few SDGs remain unaddressed such as no poverty, reduced inequalities, and peace and justice strong institution. The key activities undertaken by each of the companies to meet their sustainability agenda are summarized in the following. Their COVID-19 response plan is also given in Supplementary Table S2.

#### Johnson & Johnson highlights

Under the SDG 3, vaccine distribution and setting of new health centers under the Janssen CarePath program were key highlights. Drug innovation and remote training system were set up to facilitate training and development. Earthwards program to develop sustainable products remains a priority for Johnson & Johnson. A number of partnerships came into play to achieve the objective of developing new treatment and sustainable production (45).

#### Pfizer highlights

As part of the SDG 3, Pfizer launched the development of a digital health application and a global health innovation grant program to address primary healthcare. Education on vaccination for the community was a key highlight. Wastewater management, water recycling, and application of green chemistry aimed to address environmental issues (46).

#### Eli Lilly highlights

Eli Lilly activities in the year 2019 showed alignment with good health and well-being, quality education, gender equality, clean water and sanitation, affordable and clean energy, life on land, and building partnership. The activities related to good health and well-being included donating essential medicines, improving diabetes care, and developing the serological test for the COVID-19 pandemic (47).

#### Bayer highlights

In 2019, Bayer supported food production by providing measures for sustainable farming, distribution of high-yield seeds, integrated weed management, and crop science development. Bayer demonstrated its responsible production intent by developing computer-aided chips to reduce animal testing and hazardous chemical use in various manufacturing processes in 2019 (48).

#### GSK highlights

Under good health and well-being, GSK used a value-based approach to lower the price of medicines in 2019. In addition to it, medical treatment and vaccine distribution, special Dengue fever services, and healthcare workers training programs were set up across India and Africa. GSK continues to undertake measures to recycle water used in process of manufacturing. A number of partnership and collaboration in the area of functional genomics, vaccine development, and saving the children were done in 2019 (49).

#### Sanofi highlights

Sanofi in 2019 continued to support good health in the community by providing inactivated polio vaccine through GAVI to ~70 million children worldwide. It also provided treatment to children suffering from cancer through the Sanofi Espoir Foundation. New programs were developed to increase a number of internship opportunities for both students and employees. Constant efforts were made to increase environmental awareness and reduce greenhouse gas emissions (50).

#### AstraZeneca highlights

To promote good health and better access to healthcare, various health programs such as health heart Africa, young health, healthy lung, and mental well-being program were launched in 2019. A global workforce well-being program provided employee health coverage. To make more sustainable cities, a sustainable matter week conference was organized in 2019. Green lab program and assessment of 131 manufacturing sites using a supplier sustainability framework was also done (51).

#### Aspen highlights

Aspen is a leading pharmaceutical industry in the African continent when it comes to sustainability. To promote good health in African society, low-cost antiretroviral therapy for HIV, a special disease management program, and mental health problems advice were provided under the CSR agenda. Solar energy projects and water recycling plants were set up across manufacturing units to conserve essential natural resources (52).

#### Sun pharma highlights

Sun Pharma is a reputed Indian company in Asia and has been active in the field of sustainability reporting. Low-cost antiviral drugs, mobile health services, and *Navya* online expert opinion were major SDG 3 measures. Water conservation project and energy conservation measures were implemented to have a responsible production and consumption process (53).

#### Bharat biotech highlights

Although it is a small Indian company, it promotes sustainability culture across its units. In 2019, a regular health check-up program for employees was started along with education sessions on lifestyle diseases open to both employees and society. Basic life support training for employees was provided to make the work environment safe. Water recycling units and treatment plan have been established to avoid wastage of natural resources (54).

## Discussion

### Cross-walk among SDGs, JEE, and one health approach

*RQ1. Is there any relationship between the SDGs and development of National Action Plan for Health Security (NAPHS) based on the JEE assessment tool and One Health approach?*

The SDGs and set targets under prevent, detect, response, and other IHR-related hazards in the JEE assessment tool (21) are two separate entities as mentioned previously. Similarly, the One Health approach overarches both SDGs and elements of JEE. The cross-walk exercise in this study showed that activities under specific SDGs are directly or indirectly linked to the JEE and One Health approach. Cross-walk exercises have been used to identify priorities and overlapping tasks in the health sector and in Tanzania's multi-sectoral NAPHS (55). This exercise assisted us in identifying potential overlap between the activities required to attain the SDGs and the JEE targets. For example, the food safety target set in JEE refers to the establishment of system for surveillance and control over food contamination to avoid food wastage which relates to SDG 2 “zero hunger” and minimizing poverty. This is also directly under human and animal health of the One Health approach. Similarly, good health and well-being is a cumulative result of the JEE assessment categories IHR coordination, communication and advocacy, zoonotic disease, food safety, biosafety and biosecurity, immunization, national laboratory system, real-time surveillance, and linking public health and security authorities which is also under the triad components of the One Health approach as given in Table 4. Considering the current COVID-19 pandemic and related vaccine stigma, the issue of quality education about immunization, biosafety, infection, prevention, and control cannot be undermined in addition to coordination among various stakeholders. This shows a direct relationship between the SDGs and JEE elements under prevent category. COVID-19 zoonotic transmission relates directly to both human and animal health under the One Health approach.

For potential growth in any industry, a healthy, well-diversified workforce is essential, and it relates to both SDGs and human health component of the One Health approach triad. Natural resource conservation and responsible use remain one of the most important SDGs. It includes the use of clean water and responsible consumption of natural resources in the process of manufacturing. This is closely related to the set targets in the JEE for the surveillance of zoonotic diseases, chemical events, food safety, and all triad components of the One Health approach. According to the JEE, the goal for zoonotic disease surveillance is to prevent zoonotic disease transmission from animals to humans through contaminated water and food chain. This not only causes food scarcity and wastage, but also increases poverty and difficulty in achieving SDG 2 “zero hunger.” Hence, a comparable surveillance and response system should be in place to prevent foodborne infections. This is directly related to the One Health approach as the transmission of zoonotic diseases remains a challenge to overcome. Urbanization and industrialization are one of the contributing factors for increasing zoonotic diseases, thereby affecting both animal and environmental health under the One Health approach. Production processes often use natural resources and their by-products damage microecosystems, causing zoonotic illnesses. Pharmaceutical industries' by-products and waste are known to damage the marine flora and fauna. Pharmaceutical waste from antibiotic production and its unregulated use is the main cause of antibiotic resistance. Antimicrobial resistance remains a challenge and comes directly under the One Health approach. The JEE set targets in this reference for food safety, zoonotic diseases, and chemical events are important in achieving the SDGs. To achieve this set target, the One Health approach needs to be adopted at all levels of policymaking.

A proper national policy and partnership building is required not only to attain the SDGs but also to fulfill the targets mentioned in the JEE assessment tool. To fulfill both the SDGs and the JEE targets, there is a need for interlinkage across several sectors, such as finance and agriculture, and other stakeholders (56). The One Health approach should be adopted to include all the three aspects, namely, human, animal, and environment. This justifies that the SDGs and JEE elements complement each other under the umbrella of the One Health. The development of NAPHS is based on the results of the JEE assessment. Cross-walk exercises reveal that weak areas are linked directly or indirectly to the SDGs, and companies should be encouraged to contribute to the linked SDGs to promote increased global health security. For example, if a pharmaceutical industry is unable to manage antibiotic waste products resulting in contamination of land and water and thus increasing antibiotic resistance, national regulatory bodies should assist the industry not only with technical expertise as required to achieve the JEE–AMR target, but also with achieving the SDGs of responsible consumption and production. Similarly, the pharmaceutical industry can provide their expertise in the field of supply chain, laboratory testing, research, and development in preparation of emergency response operation during a global health crisis.

Eminent researchers such as Smith (57) and Kickbusch et al. (37) have advocated considering the EVD outbreak an inclusion of global health security as SDG 18 in the sustainability agenda. The relationship between the SDGs and global health security has been acknowledged, and the cross-walk exercise results ascertain this argument.

### Review of the sustainability reporting across the pharmaceutical companies in 2019 and COVID-19 response

*RQ2. Do the activities under the COVID-19 response plan directly or indirectly contribute to fight against the pandemic?*

Sustainability reporting among the selected 10 multinational pharmaceutical firms is comprehensive and systematic. However, there is always a scope of standardization when it comes to reporting those SDGs. The activities reported were categorized against the 17 SDGs as recommended by the UN Global Compact (25).

The COVID-19 pandemic garnered global attention, and its response planning became a priority. The 10 selected companies' action plans shared a few commonalities, such as boosting access to essential medicines and supporting diagnostic and therapeutic research and development. However, the individual companies used their area of expertise to support communities in their own peculiar way. For example, Bayer used drones to improve distribution of essential COVID-19 kits to the farmers (48). Pfizer's expertise in the development of therapeutics in particular vaccines gave it an advantage to enter into productive collaboration with BioNTech to develop the COVID-19 vaccine (46). Similarly, Eli Lilly promoted the development of biologics to treat COVID-19 and secured non-prescription availability of Insulin (47). The vaccine breakthrough by Pfizer, AstraZeneca, and Bharat Biotech is well-appreciated globally.

Even though the activities done differed significantly, all 10 selected companies focused their efforts on the SDGs. This shows that the pharmaceutical sector holds the potential to contribute to global health security.

### How can the pharmaceutical companies' sustainability activities help countries to develop NAPHS and global health security?

Cross-walk exercises show that all activities undertaken by the pharmaceutical companies under their sustainability agenda directly or indirectly contribute to fulfilling the targets in the JEE assessment tool. These activities also related to the triad components of the One Health approach as given in Table 4. This indicates the possibility of collaboration between the pharmaceutical industry and other government agencies to build a NAPHS. A common agenda for the sustainability activities that are prioritized in the JEE assessment can be developed at the national level as per country requirements. Pharmaceutical companies might be directed by national government bodies to invest their resources in sustainable activities that will enhance key areas for boosting health security. Pharmaceutical businesses also contribute a variety of skill sets through internal and external stakeholders, which can be crucial in developing a resilient healthcare system. These skills are in the area of research and development, network of associated clinicians, logistic supply network through distributors, human resources, and global network through external stakeholders. A public–private partnership with pharmaceutical industries in the area of public health can leverage the connections and skills of the companies and equally help the pharmaceutical industry to overcome their challenges in alignment of their activities with the SDGs.

This study is a beginning step in the subject of health security; more in-depth studies can be undertaken based on this study to explore the following:

(a) Sustainable development goals and health security are closely related to each other and
(b) Activities performed by the pharmaceutical industry as part of their sustainability agenda can be tailored to increase health security and fight against the pandemic.

### Recommendations for pharmaceutical companies to align with SDGs

Based on this study and previous research, we propose that pharmaceutical companies need to be more transparent, communicative, and collaborative to contribute maximum toward the SDGs. An interesting study conducted by Sinkovics and coworkers provided a matrix that can help pharmaceutical companies to classify their activities into positively, neutrally, or negatively influence particular SDGs (58). Additionally, pharmaceutical industries can further divide their activities into associative activities which means firms' involvement in achieving the SDGs through their networks, peripheral activities showing voluntary activities supporting the SDGs through voluntary actions, operational activities showing actions aligned to the SDGs, and finally embedded activities which encompass the company products in line with the objective of the SDGs.

Therefore, on the basis of this study, we propose that pharmaceutical industries should do activities in the area of unaddressed SDGs beyond SDG 3 which is good health and well-sbeing. These include quality education (SDG 4), clean water and sanitation (SDG 6), sustainable cities and communities (SDG 11), and life below water (SDG 14).

### Future contributions

This study is based on the lessons learned from the recent COVID-19 pandemic and the urgency to develop a resilient health system and be prepared for future pandemics/public health emergencies. When it comes to building robust health security, it is a well-known fact that resources are limited. Hence, this study explores how pharmaceutical industries' sustainability initiatives may be utilized or tailored to increase health security and combat pandemics. To achieve this goal, it is required to first identify whether there is any cross-link among sustainable development goals and various laid down frameworks important for health and well-being, such as the joint external evaluation tool recommended by the WHO and One Health approach. Although in the past, researchers advocated the inclusion of health as a separate SDG or applying the One Health approach globally to enhance health security; however, this study closely interlinks the SDGs, health security, and One Health. This study will be helpful not only in utilizing resources toward the development of health security but also in formulating public health policies, particularly in emergency preparedness.

### Study limitations

This study is descriptive in nature. Due to the ongoing pandemic and limited resources, only 10 companies could be included in this study. Selection bias cannot be ruled out because this study is based on voluntary disclosures of the activities and published reports. The limited literature and data are available in this domain.

## Conclusion

This study provides an insight into the activities and measures adopted by 10 multinational pharmaceutical companies under their sustainability agenda and as part of the COVID-19 response plan. An attempt to cross-link the 17 SDGs, the JEE assessment tool, and the triad components of the One Health approach has been made to show the possible future scope of integrating pharmaceutical companies' expertise in building resilient health system. This study opens a window in this area and can be helpful to various government and non-government stakeholders to consider the integration of the pharmaceutical sector to increase health security.

## Data availability statement

The original contributions presented in the study are included in the article/Supplementary material, further inquiries can be directed to the corresponding author.

## Ethics statement

This study does not include any human, patient, or animal data and there is no experimental work or clinical trials involved. It only includes the information on policy making available in the public domain. Hence, formal approval from the ethics committee is not required for this study.

## Author contributions

KS has done the conceptualization, written the original draft of the article, and addressed critical points raised by PS. Further inputs provided by SB and PS on methodology and discussion. KS is responsible for all correspondence regarding this article. All authors approved the submitted version.

## Funding

This is a self funded study.

## Conflict of interest

The authors declare that the research was conducted in the absence of any commercial or financial relationships that could be construed as a potential conflict of interest.

## Publisher's note

All claims expressed in this article are solely those of the authors and do not necessarily represent those of their affiliated organizations, or those of the publisher, the editors and the reviewers. Any product that may be evaluated in this article, or claim that may be made by its manufacturer, is not guaranteed or endorsed by the publisher.
