# Supplementary material for: The role of pharmaceutical industry in building resilient health system
Source: Front Public Health. 2022 Dec 1;10:964899. doi: 10.3389/fpubh.2022.964899 (PMC9751196; doi:10.3389/fpubh.2022.964899)
Supplement: Supplementary file 1 [file Table_1.pdf]

Supplementary Table S1: Set targets under each category in joint external evaluation tool[a], Sustainable Development Goals, One Health, India, 2019-2021

Prevent (P)	Target
(P1) National Legislation, Policy and financing	Adequate legal framework for States Parties to support and enable the implementation of all their obligations and rights made by the IHR. Development of new or modified legislation in some States Parties for the implementation of the Regulations. Where new or revised legislation may not be specifically required under a State Party's legal system, the State may revise some legislation, regulations or other instruments in order to facilitate their implementation in a more efficient, effective or beneficial manner. States Parties ensure provision of adequate funding for IHR implementation through the national budget or other mechanisms. Country has access to financial resources for the implementation of IHR capacities. Financing that can be accessed on time and distributed in response to public health emergencies, is available.
(P2) IHR Coordination, Communication and Advocacy	Multisectoral/multidisciplinary approaches through national partnerships that allow efficient, alert and response systems for effective implementation of the IHR. Coordinate nationwide resources, including sustainable functioning of a National IHR Focal Point – a national centre for IHR communications which is a key obligation of the IHR – that is accessible at all times. States Parties provide WHO with contact details of National IHR Focal Points, continuously update and annually confirm them.
(P3) Antimicrobial resistance	A functional system in place for the national response to combat antimicrobial resistance (AMR) with a One-Health approach, including:<br><br>a) Multisectoral work spanning human, animal, crops, food safety and environmental aspects. This comprises developing and implementing a national action plan to combat AMR, consistent with the Global Action Plan (GAP) on AMR.<br><br>b) Surveillance capacity for AMR and antimicrobial use at the national level, following and using internationally agreed systems such as the WHO Global Antimicrobial Resistance Surveillance System (GLASS) and the OIE global database on use of antimicrobial agents in animals.<br><br>c) Prevention of AMR in health care facilities, food production and the community, through infection prevention and control measures.<br><br>d) Ensuring appropriate use of antimicrobials, including assuring quality of available medicines, conservation of existing treatments and access to appropriate antimicrobials when needed, while reducing inappropriate use.

(P4) Zoonotic disease	Functional multisectoral, multidisciplinary mechanisms, policies, systems and practices are in place to minimize the transmission of zoonotic diseases from animals to human populations.
(P5) Food safety	Functional system is in place for surveillance and response capacity of States Parties for foodborne disease and food contamination risks or events with effective communication and collaboration among the sectors responsible for food safety.
(P6) Biosafety and biosecurity	A whole-of-government multisectoral national biosafety and biosecurity system with dangerous pathogens identified, held, secured and monitored in a minimal number of facilities according to best practices; biological risk management training and educational outreach conducted to promote a shared culture of responsibility, reduce dual-use risks, mitigate biological proliferation and deliberate use threats, and ensure safe transfer of biological agents; and country specific biosafety and biosecurity legislation, laboratory licensing and pathogen control measures in place as appropriate.
(P7) Immunization	A national vaccine delivery system – with nationwide reach, effective distribution, easy access for marginalized populations, adequate cold chain and ongoing quality control – that is able to respond to new disease threats.
**Detect (D)**	**Target**
(D1) National Laboratory system	Surveillance with a national laboratory system, including all relevant sectors, particularly human and animal health, and effective modern point-of-care<br><br>and laboratory-based diagnostics.
(D2) Real times surveillance	(1) Strengthened indicator-based and event-based surveillance systems that are able to detect events of significance for public health and health security;<br><br>(2) improved communication and collaboration across sectors and between subnational (local and intermediate), national and international levels of authority regarding surveillance of events of public health significance; and (3) improved national and intermediate level regional capacity to analyse and link data from and between, strengthened early-warning surveillance, including interoperable1, interconnected electronic tools. This would incorporate epidemiological, clinical, laboratory, environmental testing, product safety and quality, and bioinformatics data; and advancement in fulfilling the core capacity requirements for surveillance in accordance with the IHR and OIE guidelines.
(D3) Reporting	Timely and accurate disease reporting according to WHO requirements and consistent relay of information to FAO and OIE.

(D4) Workforce development	States Parties with skilled and competent health personnel for sustainable and functional public health surveillance and response at all levels of the health<br><br>system and the effective implementation of the IHR. Human resources include nurses and midwives, physicians, public health and environmental specialists, social scientists, communication, occupational health, laboratory scientists/technicians, biostatisticians, information technology (IT) specialists and biomedical technicians. There is a corresponding workforce in the animal sector of veterinarians, animal health professionals, para-veterinarians, epidemiologists, and IT specialists. The recommended density of doctors, nurses and midwives per 1000 population for operational routine services is 4.45 plus 30% surge capacity. The optimal target for surveillance is one trained (field) epidemiologist (or equivalent) per 200 000 population who can systematically cooperate to meet relevant IHR and PVS core competencies. One trained epidemiologist is needed per rapid response team.
**Respond (R)**	**Target**
(R1) Emergency Preparedness	States Parties conduct “emergency preparedness” (defined as, the knowledge and capacities and organizational systems developed by governments, response and recovery organizations, communities and individuals to effectively anticipate, respond to, and recover from the impacts of likely, imminent, emerging or current emergencies), which is a combination of planning, allocation of resources, training, exercising, and organizing to build, sustain and improve operational capabilities at national, intermediate and local or primary response levels based on strategic risk assessments. The strategic risk assessment identifies, analyses and evaluates the range of risks in a country and enables risks to be assigned a level of priority and includes analyses of potential hazards exposures and vulnerabilities, identification and mapping of available resources, and analyses of capacities (routine and surge) at the national, intermediate and local or primary levels to manage the risks of outbreaks and other emergencies. Emergency preparedness applies to any hazard that may cause an emergency and includes biological, chemical, radiological and nuclear, natural, other technological and societal hazards.
(R2) Emergency Response Operations	Countries will have a coordination mechanism, incident management systems, exercise management programmes and public health emergency operation centre (EOC) functioning according to minimum common standards; maintaining trained, functioning, multisectoral rapid response teams, and trained EOC staff capable of activating a coordinated emergency response within 120 minutes of the identification of an emergency.
(R3) Linking public health and	Country conducts a rapid, multisectoral response1 for any event of

security authorities	suspected or confirmed deliberate origin, including the capacity to link public health and law enforcement, and to provide timely international assistance.
(R4) Medical countermeasures and personnel deployment	National framework for: transferring (sending and receiving) medical countermeasures, and public health and medical personnel from international partners during public health emergencies; and procedures for case management of events due to IHR relevant hazards.
(R5) Risk communication	States Parties use multilevel, multisectoral and multifaceted risk communication capacity for public health emergencies. Real-time exchange of information, advice and opinions during unusual and unexpected events and emergencies so that informed decisions to mitigate the effects of threats, and protective and preventative action can be made. This includes a mix of communication and engagement strategies, such as media and social media communications, mass awareness campaigns, health promotion, social mobilization, stakeholder engagement and community engagement.
**Other IHR related hazards (O)**	**Target**
(O1) Points of entry	States Parties designate and maintain core capacities at international airports and ports (and where justified for public health reasons, a State Party may designate ground crossings) that implement specific public health measures required to manage a variety of public health risks.
(O2) Chemical entry	States Parties with surveillance and response capacity for chemical risks or events. This requires effective communication and collaboration among the sectors responsible for chemical safety, industries, transportation and safe disposal, animal health and the environment.

Note:
[a]The reference of this table is (21) in the reference list: World Health Organization. Joint external evaluation tool: International Health Regulations 2005 (2016). Available at: https://apps.who.int/iris/handle/10665/204368 [Accessed January 25, 2021]
