# Supplementary material for: The role of pharmaceutical industry in building resilient health system
Source: Front Public Health. 2022 Dec 1;10:964899. doi: 10.3389/fpubh.2022.964899 (PMC9751196; doi:10.3389/fpubh.2022.964899)
Supplement: Supplementary file 2 [file Table_2.pdf]

Supplementary Table S2: COVID-19 response plan of ten pharmaceutical companies, Sustainable Development Goals, One Health, India, 2019-2021

S.No.	Name of the company	COVID-19 response
1	Johnson & Johnson[a] (45)	• Access to medicine - ensuring critical medicine supply chain<br>• Employee Safety - training & development of SOPs for infection prevention control<br>• Development of COVID vaccine & other therapeutics<br>• PRISMA health device distribution to allow single ventilator to use by two patients at the same time.
2	Pfizer[b] (46)	• Adopted five-point plan which included sharing tool and insight available on various platform, creating a SWAT team, sharing drug development expertise, offering large scale production and providing support to Center for Disease Control (CDC).<br>• Collaborated with BioNTech to develop a mRNA-based vaccine known as BNT162b2.
3	Eli Lilly[c] (47)	• Adopted five key elements approach which included maintaining access to medicine especially Insulin, ensuring employee safety and support communities by providing education and rapid testing.
4	Bayer[d] (48)	• Developed 3R approach that stands for response, resilience, and recovery.<br>• Introduced special programs to support farmers. Special kits with essential medicine, pesticides and vital seeds were distributed at no cost to ensure that agricultural productivity remains unaffected.
5	GSK[e] (49)	• Two new collaborations to accelerate development of vaccines.<br>• First collaboration with coalition for epidemic preparedness in Innovations (CEPI) and second with china-based Clover biopharmaceuticals.
6	Sanofi[f] (50)	• Collaborated with US department of health to accelerate development of vaccine.<br>• Clinical trial program for testing Sailumab efficacy in severe COVID -19 patient
7	AstraZeneca[g] (51)	• Ensured access to medical products and employee safety.<br>• Donation of PPE (personal protective equipment) in 49 countries and an accelerated program for vaccine

		development in collaboration with University of Oxford<br>• Development of neutralising long acting antibody to treat COVID-19 patient<br>• Special arrangements were made to launch COVAX in collaboration with other partners like GAVI for rapid distribution of the vaccine globally
8	Aspen[h] (52)	• Securing access to the essential medicines, training employees to follow infection, prevention, and control guidelines to avoid the spread of the virus<br>• Assisting in vaccine production candidate Ad26.COV2-S in collaboration with Johnson & Johnson.
9	Sun Pharma[i] (53)	• Medicines and hand sanitisers were donated to various hospitals and healthcare units<br>• Organised awareness programs on COVID-19 in the society<br>• Distributed food packets to rural communities
10	Bharat Biotech[j] (54)	• Developed COVAXIN

Note:

[a]Please see reference number (45) in the reference list:Johnson & Johnson. 2019 Health for Humanity Report, Progress in Sustainability, Our COVID-19 Efforts (2020). Available at: https://healthforhumanityreport.jnj.com/united-in-defeating-covid-19. https://healthforhumanityreport.jnj.com/_document/2019-heath-for-humanity-report-johnson-johnson?id=00000172-a8f8-dff3-a9fa-acfda52c0000. https://www.jnj.com/coronavirus [Accessed November 19, 2020].

[b]Please see reference number (46) in the reference list: Pfizer. Pfizer 2019 Annual Review Breakthroughs that change patients' lives, COVID-19 INFORMATION AND RESOURCES (2020). [cited 2021 Feb 10]. Available at: https://www.pfizer.com/sites/default/files/investors/financial_reports/annual_reports/2019/assets/pfizer-2019-annual-review.pdf, https://www.pfizer.com/health/coronavirus [Accessed February 10, 2021].

[c]Please see reference number (47) in the reference list: Eli Lilly. 2019 United Nations Global Compact Communication on Progress Report, COVID-19 Response (2020). Available at: https://assets.ctfassets.net/srys4ukjcerm/1NNksnwvNZGqMMXOO4wcsl/3754b6ecf7ddf7b140ef2b8744fff7c5/United_Nations_Global_Compact_Communication_on_Progress_Report.pdf, https://www.lilly.com/news/stories/2021-coronavirus-covid19-global-response [Accessed November 19, 2020].

[d]Please see reference number (48) in the reference list: Bayer. 2019 Sustainability Report, Update on COVID-19 (2020) [cited 2020 Dec 5]. Available at: https://www.bayer.com/sites/default/files/2020-12/bayer-ag-sustainability-report-2019_5.pdf, https://www.bayer.com/en/covid-19/coronavirus-covid-19-update-news. [Accessed December 5, 2020].

[e]Please see reference number (49) in the reference list: GSK. 2019 ESG Performance Summary, Our response to COVID-19 (2020). Available at: https://www.gsk.com/media/5886/esg-performance-summary-2019.pdf. https://www.gsk.com/media/5978/coronavirus_factsheet_2.pdf [Accessed December 14, 2020].

[f]Please see reference number (50) in the reference list: Sanofi. 2019 Corporate Social Responsibility, Our response to COVID-19 (2020). Available at: https://www.sanofi.com/-/media/Project/One-Sanofi-Web/Websites/Global/Sanofi-COM/Home/common/docs/our-responsibility/documents-center/factsheets-pdf4-2020/Sanofi-Chapter4 EN_accessible.pdf?la=en&hash=25B33FE52F73FFF6ABD29D6D70431068, https://www.sanofi.us/en/about-us/our-stories/our-response-to-covid-19 [Accessed November 10, 2020].

[g]Please see reference number (51) in the reference list: AstraZeneca. Sustainability Report, 2020 COVID-19 pandemic actions (2020). Available at: https://www.astrazeneca.com/content/dam/az/Sustainability/2020/pdf/Sustainability_Report_2019.pdf.https://www.astrazeneca.com/content/dam/az/Sustainability/2021/pdf/COVID-19_pandemic_actions.pdf [Accessed January 10, 2021].

[h]*Please see reference number (52) in the reference list: Aspen Pharmacare Holdings Limited. Sustainability Data Supplement 2019, Our Response to COVID-19 (2020). Available at: https://www.aspenpharma.com/wp-content/uploads/2019/10/Aspen-Sustainability-Data-Supplement-2019.pdf.https://www.aspenpharma.com/our-response-to-covid-19/ [Accessed January 10, 2021].*

[i]*Please see reference number (53) in the reference list: Sun Pharma. 2019-2020 Annual Report Business Responsibility Report, COVID-19 (2020). Available at: https://www.sunpharma.com/sites/default/files/annual/SPIL-AR2019-20-BusinessResponsibilityReport.pdf, https://www.sunpharma.com/covid-19/news- and-updates. [Accessed November 08, 2020].*

[j]*Please see reference number (54) in the reference list: Bharat Biotech. COVID-19. Available at: https://www.bharatbiotech.com. [Accessed September 15, 2020].*
